# SIADH versus adrenal insufficiency: a life-threatening misdiagnosis

**DOI:** 10.1186/s13052-019-0614-1

**Published:** 2019-02-06

**Authors:** Stefano Pintaldi, Angela Lora, Katy Vecchiato, Andrea Taddio, Egidio Barbi

**Affiliations:** 1 University of Trieste, Piazzale Europa, 1, 34127 Trieste, Italy; 2 Department of Pediatrics, Institute for Maternal and Child Health-IRCCS “Burlo Garofolo”, Trieste, Italy; 3 Forensic & Neurodevelopmental Sciences, King’s College London, London, England, UK

**Keywords:** Adrenal insufficiency, Primary adrenal insufficiency, Addison disease, Syndrome of inappropriate antidiuretic hormone secretion, Hyponatremia, Gastroenteritis

## Abstract

**Background:**

Primary adrenal insufficiency (PAI) in children is an uncommon but severe condition which can be either inherited or acquired. It consists in clinical manifestation of defective production or ineffective action of endogenous glucocorticoids; deficiency in mineralocorticoids and adrenal androgens may coexist. Diagnosis of PAI in children and young people can be challenging; while adrenal crisis (acute decompensation) is a life-threatening condition, with patient presenting with characteristic features of hypoglycemia, hypotension, collapse and coma, chronic adrenal insufficiency may present with vague and non-specific symptoms, making the diagnosis more difficult.^1^ Gastroenteritis and Syndrome of Inappropriate Secretion of Antidiuretic hormone (SIADH) are the most frequent reported misdiagnosis in patients with adrenal insufficiency (AI). While intravenous fluid replacement in the suspect of a gastroenteritis would be beneficial, a SIADH misdiagnosis can be harmful since the treatment of this condition is based primarily on fluid restriction.

**Case presentation:**

We report the case of a child admitted to the emergency department whose condition was ultimately diagnosed as autoimmune adrenal insufficiency after few hours of inappropriate fluid restriction following a SIADH misdiagnosis.

**Conclusions:**

AI is a rare condition in children and the diagnosis can be challenging. A missed diagnosis of AI or an inadequate treatment may cause severe complications, especially if a SIADH is erroneously diagnosed. Emergency physicians and pediatricians should be familiar with this diagnosis to enhance early recognition of this potentially life-threatening condition.

## Background

Primary adrenal insufficiency (PAI) in children is an uncommon but severe condition which can be either inherited or acquired. It consists in clinical manifestation of defective production or ineffective action of endogenous glucocorticoids; deficiency in mineralocorticoids and adrenal androgens may coexist. Diagnosis of PAI in children and young people can be challenging; while adrenal crisis (acute decompensation) is a life-threatening condition, with patient presenting with characteristic features of hypoglycaemia, hypotension, collapse and coma, chronic adrenal insufficiency may present with vague and non-specific symptoms, making the diagnosis more difficult [1]. Gastroenteritis and Syndrome of Inappropriate Secretion of Antidiuretic hormone (SIADH) are the most frequent reported misdiagnosis in patients with adrenal insufficiency (AI). While intravenous fluid replacement in the suspect of a gastroenteritis would be beneficial, a SIADH misdiagnosis can be harmful since the treatment of this condition is based primarily on fluid restriction.

## Case presentation

A 12 year-old boy Caucasian boy was admitted during his summer holydays to a pediatric emergency department with repeated vomiting, malaise, excessive thirst, dizziness on standing and one episode of syncope after drinking 1 liter of water.

His past history was remarkable for a similar episode 3 weeks before: after repeated vomiting he had been admitted to the local hospital and a marked hyponatraemia (124 mEq/L) was found. He was diagnosed with gastroenteritis, hyponatraemia was corrected with normal saline infusion and the boy was discharged. He eventually remained symptom-free until the actual episode.

At admission no fever, diarrhea, change in urinary output or change in weight were reported. Physical examination showed an apyretic, eupnoic, asthenic boy, with a tanned skin color. Heart rate was 63 bpm, blood pressure was 98/62 mmHg and SaO2 was 100%.

Laboratory tests showed hyponatraemia (121 mEq/L), hypochloraemia (86 mEq/L) and mild hyperkalaemia (5,91 mEq/L) with low plasmatic osmolarity (248 mOsm/Kg). Urinary sodium was 163 mEq/L, potassium 48 mEq/L, chlorine 119 mEq/L, with high urinary osmolarity (896 mOsm/L; 300–900). Blood and urinary glucose, white cell count, blood gas analysis and renal function were normal.

A syndrome of inappropriate secretion of antidiuretic hormone (SIADH) was suspected and fluid restriction with two-thirds of the standard maintenance rate of normal saline was carried out.

After 4 hours hyponatraemia worsened (119 mEq/L) despite fluid restriction, while asthenia and inability to stand upright persisted, thus posing the suspect of adrenal insufficiency.

Low levels of cortisol (7.95 mcg/dl; 6.2–19.4 reference range), marked ACTH increase (> 1250 pg/mL), high levels of renin (> 500 mUI/ml) and low levels of aldosterone (3.1 ng/dL; 3–30 reference range) confirmed the diagnosis of adrenal insufficiency. Intravenous hydrocortisone treatment was started at the dose of 100 mg/day, then decreased to 50 mg each 6 h/day; oral daily fludrocortisone, 0.15 mg, was associated. Maintenance therapy with oral hydrocortisone was introduced after 3 days, while electrolyte and ACTH values returned within normal limits in 48 h. The patient presented two further episodes of symptomatic orthostatic hypotension in the first 72 h after diagnosis and recovered in the following days. Anti-adrenal antibodies tested positive, confirming autoimmune adrenalitis (Addison disease).

## Discussion

In pediatric patients AI is a rare condition and its true incidence is unknown; while more than 80% of cases of PAI in adults are autoimmune disorders, the most frequent cause of PAI in children is Congenital Adrenal hyperplasia (CAH) mainly due to 21-hydroxylase deficiency*.* Autoimmune adrenalitis is the second leading cause of PAI in young people, accounting for 12.7% of all cases, with prevalence increasing during the second half of the second decade of life [2]. Although autoimmune Addison disease most often occurs sporadically, it can occur as a component of autoimmune polyendocrinopathy syndromes, consisting of a constellation of disorders such as candidiasis and autoimmune conditions (hypothyroidism, hypoparathyroidism, ovarian failure, vitiligo, gastritis, type 1 diabetes mellitus, hepatitis) which should be suspected and ruled out in presence of acquired adrenal insufficiency in children [2].

Diagnosis of AI in children and young people can be challenging, especially for the chronic form of the disease, as it presents with vague and non-specific symptoms. [1]

Clinical presentation of chronic AI comprise fatigue, weight loss, nausea, vomiting, abdominal pain, salt craving, muscle and joint pain. More specific signs are hypotension and skin hyperpigmentation; the latter is generally more prominent on mucosae and on sun-exposed areas or over pressure points such as elbows and knees, and result from enhanced activation of skin melanocortin 1 receptors [MC1R]. In congenital hyposurrenalism skin hyperpigmentation can appear as early as at the end of the first month of life, but it usually appears after 4 months, hence in patients with recent onset disease, these changes in pigmentation may not be present [1, 3].

Hyponatraemia is the most common laboratory abnormality observed in PAI (90% of cases), followed by hyperkalaemia (50% of cases) and hypoglycemia (30% of cases) [4].

In our patient the initial diagnosis of SIADH seemed unlikely due to the presence of syncope and dizziness on standing, increased thirst, no weight gain and normal urinary output.

Of note, absence of hyperkalaemia and hypoglycemia does not rule out adrenal insufficiency, so initial presentation with hyponatraemia and high urinary sodium may actually mimic SIADH [1, 5].

Gastroenteritis and SIADH are the most frequent reported misdiagnosis in cases of AI. While intravenous fluid replacement in the suspect of a gastroenteritis would be of benefit, fluid restriction due to a SIADH misdiagnosis can be harmful and even potentially life-threatening.

Intravenous hydrocortisone sodium succinate is urgently required for therapy. The single dose is 25 mg for infants and toddlers, 50 mg for children (3–12 years old), 100 mg for older children. After this, the same total amount must be given in divided doses at 6 hourly intervals for the first 24 h [6].

An intravenous solution of 5% glucose in 0.9% saline should be administered to correct hypoglycaemia and hyponatraemia. A rapid correction of severe hyponatraemia may causes pontine myelinolysis and even death [6, 7].

After the acute phase is solved, lifelong maintenance oral therapy with hydrocortisone (8–12 mg/m^2^ daily) and Fludrocortisone (0.05–0.2 mg daily) if aldosterone deficiency is present, is required.

Intramuscular Hydrocortisone may be necessary, increasing by two or threefold the dose during episodes of stress, if the patient is not able to take the treatment orally.

## Conclusion

AI is a rare condition in children and the diagnosis can be challenging. A missed diagnosis of AI or an inadequate treatment may cause severe complications, especially if a SIADH is erroneously diagnosed. Hence emergency physicians and pediatricians should be familiar with this diagnosis to enhance early recognition of this potentially life-threatening condition.

## Abbreviations

AI: Adrenal Insufficiency
CAH: Congenital Adrenal Hyperplasia
PAI: Primary Adrenal Insufficiency
SIADH: Syndrome of Inappropriate Antidiuretic Hormone Secretion
